# CellSegm - a MATLAB toolbox for high-throughput 3D cell segmentation

**DOI:** 10.1186/1751-0473-8-16

**Published:** 2013-08-09

**Authors:** Erlend Hodneland, Tanja Kögel, Dominik Michael Frei, Hans-Hermann Gerdes, Arvid Lundervold

**Affiliations:** 1 Department of Biomedicine, University of Bergen, Bergen, Norway; 2 Department of Radiology, Haukeland University Hospital, Bergen, Norway

**Keywords:** Automated analysis, Cell segmentation, CellSegm, High-throughput, Nucleus staining, Surface staining

## Abstract

The application of fluorescence microscopy in cell biology often generates a huge amount of imaging data. Automated whole cell segmentation of such data enables the detection and analysis of individual cells, where a manual delineation is often time consuming, or practically not feasible. Furthermore, compared to manual analysis, automation normally has a higher degree of reproducibility. CellSegm, the software presented in this work, is a Matlab based command line software toolbox providing an automated whole cell segmentation of images showing surface stained cells, acquired by fluorescence microscopy. It has options for both fully automated and semi-automated cell segmentation. Major algorithmic steps are: (i) smoothing, (ii) Hessian-based ridge enhancement, (iii) marker-controlled watershed segmentation, and (iv) feature-based classfication of cell candidates. Using a wide selection of image recordings and code snippets, we demonstrate that CellSegm has the ability to detect various types of surface stained cells in 3D. After detection and outlining of individual cells, the cell candidates can be subject to software based analysis, specified and programmed by the end-user, or they can be analyzed by other software tools. A segmentation of tissue samples with appropriate characteristics is also shown to be resolvable in CellSegm. The command-line interface of CellSegm facilitates scripting of the separate tools, all implemented in Matlab, offering a high degree of flexibility and tailored workflows for the end-user. The modularity and scripting capabilities of CellSegm enable automated workflows and quantitative analysis of microscopic data, suited for high-throughput image based screening.

## Background

Cell segmentation is the process of separating every imaged cell from the background and from other cells. Automated cell segmentation is useful for the analysis of cells imaged by fluorescence microscopy, both in terms of objectivity and reduced work load. It enables the automatic quantification of cell characteristics for a large number of cells in 3D. A whole cell segmentation can provide information affiliated with individual cells in the sample. Examples of valuable cell characteristics that can be monitored are volume, shape, signal distribution, neighbourhood relations and cell movements over time. Automated analysis should be more objective than manual analysis, and thereby enhances reproducibility. It allows the processing of a huge number of data sets that otherwise would be difficult to process either due to lack of human resources or shortcomings of human perception in 3D and time. For example, it has the ability to detect fine and subtle changes in cell morphometry between experimental conditions, and thus can distinguish between characteristics that are otherwise not easily revealed by visual inspection.

A cell segmentation can be applied to *unstained* cells [1-4]. This approach minimizes the disturbance of live cells due to the lack of chemical influence of a dye and due to a reduction of phototoxicity. The segmentation is mostly successful and extremely advantageous for single cells, however, the boundaries are not easily captured for densely clustered and unstained cells. Another option is a staining of the *cytoplasm*[5,6]. A segmentation of cytoplasmically stained cells is highly useful for single cells, and for estimating the overall cell volume of all the cells. However, for densely packed cells, this method has a substantial risk of merging single cells into doublets, triplets or even larger clusters, due to the lack of a clearly perceptible signal defining the plasma membranes between adjacent cells. As a further alternative, a whole cell segmentation of highly clustered cells can be obtained by the expression of a DNA encoding a fluorescently tagged membrane marker protein, or a dye/antibody staining of the plasma membrane or the cell surface [7-9]. Such a *surface staining* defines the outline of every cell in the image, or of a specific subset of cells expressing the marker. It is a substantial advantage compared to a cytoplasmic staining if such a staining includes the membranes separating adjacent cells.

To date, several software solutions for specialized cell segmentation have been established, and are under continuous development. For example, the widely utilized software suite CellProfiler enables the analysis of cells, with corresponding cell count, measurements of volume and protein levels, and also the analysis of more complex morphological tasks like cell or organelle shape and sub-cellular patterns [10]. The algorithmic workflow is illumination correction, cell identification based on fluorescence, and measurements of cellular features. It is an open-source project where all users can contribute by adding new modules. This clever system drives the development of numerous algorithms for open use, enabling researchers to share specialized pipelines and to reproduce the work of colleagues. However, CellProfiler was originally developed for the analysis of 2D images, and has limitations for true 3D analysis. Further examples of related software are: (i) The OMAL toolbox [11] is a MATLAB-based software tool for the automated and manual segmentation of cells and cell nuclei. It also enables the analysis of spatial distribution of FISH signals in interphase nuclei; (ii) The Mosaic group published a free MATLAB tool for the segmentation and tracking of phase-contrast movies [12]; (iii) LSDCAS is an automated system for live cell imaging and identification of cells in phase contrast images [13] or by fluorescent microscopy [14]; (iv) The free software CellTrack was developed for the segmentation and tracking of cells in phase contrast images [1]. There are also commercial programs available, as listed in Table 1. The commercial software packages are typically tailored for the pharmaceutical industry, and are also provided as binary, executable code only. Despite broad functionality and user friendliness of these packages, they often have shortcomings regarding applications in a research environment, which is demanding more flexibility as comes with programmability. Alternatively, tailored software solutions can be programmed locally where the biological demands for quantitative analysis originate.

**Table 1:** Cell segmentation software tools

**Software tool**	**Developer**	**Com**	**Website**
CellProfiler	Broad Institute	No	http://www.cellprofiler.org
OMAL toolbox	Frederick National Lab	No	http://ncifrederick.cancer.gov
Mosaic software	Mosaic group	No	http://www.mosaic.ethz.ch/Downloads/phasecontrast
LSDCAS	University of Iowa	No	http://www.uihealthcare.org/otherservices.aspx?id=21022
CellTrack	Middle East	No	db.cse.ohio-state.edu/CellTrack
	Technical University		
icy	Institut Pasteur	No	icy.bioimageanalysis.org
CyteSeer	Vala Sciences	Yes	http://www.valasciences.com/software/id/cyteseer
Cellomics	Thermo Scientific	Yes	http://www.cellomics.com
Acumen	TTP LabTech	Yes	http://www.ttplabtech.com
Epigenetics Target	Evotec	Yes	http://www.evotec.com
Profiling			
IN Cell Investigator	GE Healthcare	Yes	http://www.biacore.com
Harmony	PerkinElmer	Yes	http://www.perkinelmer.com
CellScan LS	Imstar	Yes	http://www.imstarsa.com
iCyte	CompuCyte	Yes	http://www.compucyte.com

Com = commercial.

For high-throughput, image based biological research we envisioned an easily applied, fully automated, highly accurate tool for cell segmentation. Therefore, we developed CELLSEGM, which proved to be very powerful in terms of correctly defining cell volumes. CELLSEGM is primarily a tool for segmentation of surface stained cells, being more powerful than a segmentation of cytoplasmically stained cells due to the signal present between adjacent cells. To improve the segmentation quality we additionally stained and imaged cell nuclei and used those images to generate seeds to be used as markers in the watershed segmentation. Similarly, in Han et. al [15], the membrane between adjacent cells was fluorescently labelled, and the stained nuclei, a Radon transform, iterative voting and points of saliency were used to detect structures of radial symmetry. In CELLSEGM, the segmentation process is accomplished by the watershed transform with no assumptions on symmetry. The segmentation of clustered nuclei itself was addressed in many publications [6,16-19]. This process can either be a stand-alone application or it can be integrated into a whole cell segmentation, as in CELLSEGM.

When a cell segmentation is achieved, a large range of cell features can be extracted from the data set. Such post-segmentation analysis can detect and quantify differences with respect to cell volume, shape and morphology, signal distribution, and other cell features of interest. Since life-science researchers rarely are also highly educated programmers, the segmentation program should be compatible with an easily accessible post-processing module where desired parameters can be extracted and analyzed after segmentation. The cell segmentation can be realized in CELLSEGM, and the scientists can design the post-processing module by themselves or in collaboration. This enables flexible and targeted solutions to individual projects. The potential for sharing post-processing modules between scientists in terms of reproducibility is huge. Sharing those modules can simplify and accelerate the evaluation of many microscopical studies. Our choice of MATLAB as the platform for CELLSEGM is due to the flexible and manageable environment in terms of syntax and a large library of built-in functions. A tailored parameter tuning as well as implementation of post-processing modules are easily achieved in the MATLAB environment.

In the light of the recent advancement of microscopical techniques with a broader application in both basic research and clinical diagnosis, this program can offer a significant contribution to robust data analysis/diagnosis, and thereby reduce bias introduced by manual sample evaluations. Additionally, this can potentially increase the comparability of pathological evaluations between clinics. In the next section we describe our cell segmentation tool CELLSEGM with examples of possible applications.

## Design principles and workflows

### Design of CELLSEGM

**Figure 1:**
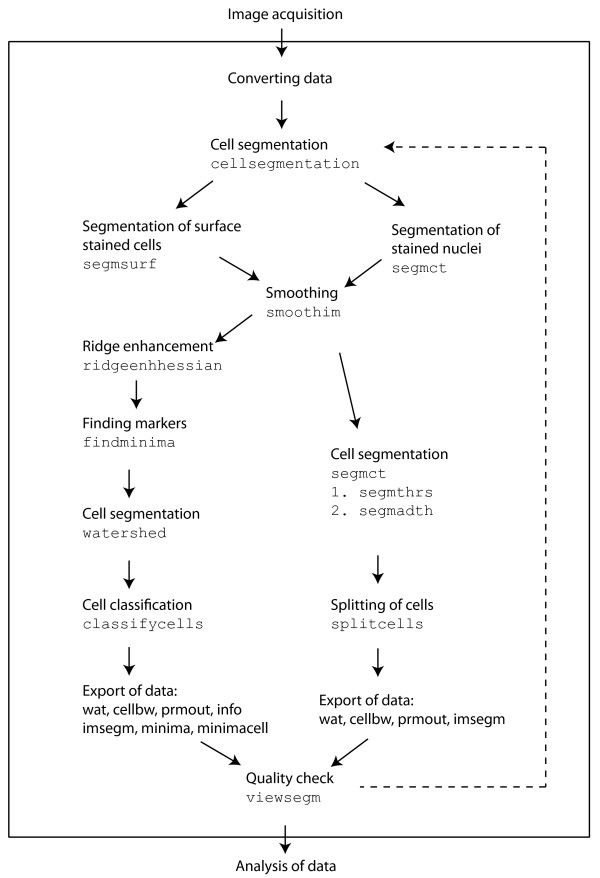
**Software design of ****CELLSEGM****.** The solid box surrounds the processing steps occuring in CELLSEGM, from image conversion until the post-analysis of the segmentation data. The batch processing cellsegmentation is the tool for cell segmentation of high-throughput data. The quality of the resulting segmentation can be assessed in viewsegm, and the processing chain can be restarted on demand (dashed line) with other parameter settings. The separate functions (m-files) can also be executed independently.

CELLSEGM is a MATLAB based command line tool for segmentation of surface stained cells, designed towards scripting and application in high-throughput experiments. The program suite accounts for all processing steps from converting the raw microscopic image files to execution of the final cell segmentation, and enables different workflows (cf. Figure 1, where the main processing steps are listed). The software suite is divided into separate modules for smoothing, ridge enhancement, finding markers, segmentation, classification, and export of data. These modules are combined in various ways in the batch processing tool cellsegmentation, where either a segmentation of surface stained cells (segmsurf) or of stained nuclei (segmct) is performed. The separate tools can also be executed from the command line in MATLAB. After segmentation, the obtained results can be quality checked using viewsegm. An unsatisfactory segmentation can be improved by parameter tuning and a re-calculation.

Currently, CELLSEGM does not support further post-processing of the segmented cells. Analysis of the cell phenotype needs to be accomplished by other software tools, or by in-house programming tailored for a specific task or project. To facilitate post-segmentation analysis or other functionality and algorithmic improvements, users of CELLSEGM are encouraged to contribute and share their code on the website of CELLSEGM.

### Implementation

All algorithmic tools in CELLSEGM are implemented in MATLAB and shared as open-source on the website (http://www.cellsegm.org) under a GNU General Public License licence. The program will run on Windows, Linux and Mac OS X platforms where MATLAB (≥ R2007b) and the MATLAB IMAGE PROCESSING TOOLBOX are installed. A speed-up can be achieved for selected parts in CELLSEGM by the use of the commercial package JACKET (http://www.accelereyes.com), a software solution for GPU computing.

### Installation and structure

CELLSEGM is installed by placing the m-files in a suitable directory and running startupcellsegm for setting the path. Additionally, the bfconvert library must be installed prior to converting raw data files into analyzeable image format (MATLAB (.mat) or tagged image format (.tif)). Consult http://www.loci.wisc.edu/bio-formats/downloads for download and further instructions.

**Figure 2:**
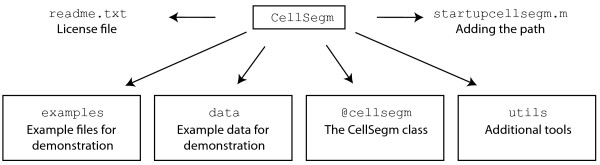
**Files and folders (inside rectangles) connected to ****CELLSEGM****.** The BDA license file (readme.txt) defines the legal rights and the startup file (startupcellsegm.m) is used for setting the path in MATLAB to enable CELLSEGM. There are four folders at the highest level, containing example files used in this work for demonstration, example data loaded by the example files, the mfiles contained in the class @CellSegm, and one folder containing additional utility files necessary for CELLSEGM.

The files connected to CELLSEGM are organized as shown in Figure 2. There are four folders, one containing the core m-files to run CELLSEGM, contained in the MATLAB class @cellsegm, one folder containing the example files from this report, one folder with example data used by the example files, and one utility folder with additional helper tools necessary to run CELLSEGM. Additionally, there are two single files, the license file readme.txt and the startup script startupcellsegm.m for setting the path in MATLAB. For executing a function in @cellsegm, always type cellsegm.myfunc.

### Usage and help

A link to this report is available on the webpage of CELLSEGM, and represents a major documentation for the usage of the software. In connection to every m-file in CELLSEGM, one can type help myfile in the MATLAB command window to see a help description for that specific tool.

### Image formats

CELLSEGM supports the use of image formats within Bio-Format (http://loci.wisc.edu/software/bio-formats), a Java library for reading and writing life sciences image formats. Using this library including BFCONVERT, CELLSEGM can be applied to .lif files. The raw data files must be exported to either image .tif files or MATLAB data files .mat. The .tif format is in particular useful for visualization using standard tools. The various channels are stored sequentially in the .tif files, first channel, then plane. In the MATLAB format, the channels are stored in the fourth dimension, thus becoming a 4D array. When using the .mat format, the conversion of raw data creates a sequence of image files with the naming stack1.mat, stack2.mat and so forth. Each of these files has two variables, im, the raw image, and h, the voxel size in micrometer, acquired from the raw data files. The function readbioformat converts the raw data to either .mat and .tif format, or only to .mat. It takes one argument, the name of the .lif file. The.mat format must at all times be present for the subsequent analysis. An example of raw data conversion using readbioformat is shown in Example 1. Be aware that this example will not run successfully with the current arguments as there are no.lif files contained in the CELLSEGM package.

*Example 1.*readlif

### Biological sample preparation and image acquisition

Since sample preparation and image acquisition are indispensable prerequisites and their proper execution is critical, we mention some of the pitfalls we experienced. We chose wheat-germ-agglutinin-Alexa-Fluor-488 conjugate (WGA-AF-488) as a plasma membrane staining. WGA-AF-488 is a lectin that binds components on the plasma membrane, which are also biologically internalized. Additionally, it attaches in a reversible manner and therefore diffuses into fixed cells within days. Both can result in, for our purpose undesired, bright staining of intracellular membranes, mainly of vesicular origin and the nuclear envelope. Those membranes are recognized by the segmentation software and can lead to false definitions of cell borders, often in the perinuclear regions of high vesicle density. In order to reduce the negative effects of biological uptake on the segmentation, one possibility is immediate imaging within 30 min after adding the dye. Alternatively, a fixation of the cells can be applied before and after the staining procedure. If the scope of the project requires image acquisition over many hours or even days due to large amounts of samples, we recommend fixation of the cells both before (to avoid biological uptake) and after the surface staining (to avoid diffusion of the staining). Another challenge lies in the fact that the Hoechst staining emission curve overlaps with the emission curve and detection range of WGA-AF-488. For optimal results, the two channels can be acquired sequentially, with the drawback of doubling the acquisition time. Alternatively, a computational dye-separation can be applied. However, we obtained good results by smoothing the channels and subtracting the Hoechst channel from the surface staining instead. For the imaging of the WGA-AF-488, it is important to carefully adjust the laser power/detection gain according to the following instructions. First, the plasma membrane-signal needs to provide sufficient contrast to other areas and display good continuity. Second, excess out-of range signal at the upper end of the intensity scale needs to be avoided, since a broad homogeneous rim with the highest possible gray-scale value (white) will lead to a placement of the cell border on the inner rim of the exaggerated membrane-image and thereby reduce the cell volume. Finally, the starting plane of 3D stacks must be carefully chosen to be the first clearly visible surface of cells, and not the poorly stained regions containing much reflected light at the substrate level, since focal planes lacking plasma membrane signal can cause the automated cell detection to fail.

## Results - Basic principles and CELLSEGM functions

### Command line based parameter settings

Proper specification and adjustment of parameters is of major importance and follows strict rules in CELLSEGM. The main routines can take an optional argument prm, a struct array defining allowable parameters. The application of prm will override the default settings in the file. Default settings are specified in the help function of each routine.

### Cell segmentation

The main processing aim of CELLSEGM is to obtain a reliable whole cell segmentation of the cell objects, meaning the mapping of every voxel as belonging to an individual cell or background. There are currently two main segmentation threads available, suitable for either segmentation of surface stained cells (segmsurf) or stained nuclei or cytoplasmically stained cells (segmct). CELLSEGM has not been tested for a cell segmentation in transmission light microscopy images. Two preprocessing steps are applied to the *segmentation image*, here defined as the input channel used for segmentation (surface or cytoplasmic stain), and to the *nucleus image* in the cases where it is defined. The optional parameters are specified in the prm struct as input to segmsurf or segmct. The preprocessing steps below are common for both segmentation threads.

### Illumination correction

The first processing step is illumination correction of the segmentation image in order to remove slowly varying intensities across the image that can influence significantly the performance of subsequent algorithms, in particular thresholding. It is accomplished by a top-hat filtering, and can be either off (prm.illum = 0, *default*) or on (prm.illum = 1).

### Smoothing

A smoothing of the segmentation image is normally advised to connect cell structures that are inhomogeneously stained and therefore incorrectly disrupted. An anisotropic smoothing algorithm is recommended instead of an isotropic since the anisotropic approach better preserves edges and ridges in the image by smoothing along the observable structures and not perpendicular. Several smoothing operations are available in CELLSEGM via the routine smoothim with different usability for various tasks. All methods in smoothim allow a 2D planewise smoothing which is normally faster and successfull, prm.planewise = 1. The choice of method in smoothim is controlled by the parameter method, given as input to smoothim, with the following options: Coherence enhancing diffusion (method = ’ced’): Partial differential equation (PDE) based anisotropic filter [20], suitable for surface stained cells. The code for 3D coherence enhancing diffusion is based on numerical computation of the eigenvalues and eigenvectors, and is therefore slower than the analytical approach present for 2D data. Directional coherence enhancing diffusion (method = ’dirced’, *default*): Mathematical morphology based anisotropic filter [21], suitable for surface stained cells. This option has a GPU version with significant speedup. However, this requires Jacket for Matlab to be installed. Edge enhancing diffusion (method = ’eed’): PDE based anisotropic filter [22], suitable for cytoplasmically stained cells and stained nuclei. Gaussian smoothing (method = ’gaussian’): Morphological filter, based on the built-in MATLAB functions imfilter (2D) and smooth3 (3D). This option is suitable for general smoothing operations using small filter radius and low standard deviation. Otherwise, the smoothing will dominate and detailed information is suppressed.

The subroutine smoothim can be executed as a stand-alone tool (specified by method) but also from inside the processing chain for segmentation (for instance specified by prm.segmsurf.smoothim.method). The segmentation is applied after these inital pre-processing steps, by either segmsurf or segmct. The syntax of the main tool is only presented with the mandatory number of arguments. Other options are described in the helper function of each separate tool.

### Segmentation of surface stained cells - segmsurf

The approach for segmentation of surface stained cells relies on a high signal on the cell boundaries, arising from the application of a fluorescent dye. The segmentation of surface stained cells is accomplished by a marker-based watershed segmentation in segmsurf, requiring three mandatory input arguments.

Syntax: segmsurf(im, minv, maxv)im: ${\text{double}}_{n_{x}\times n_{y}\times n_{z}}$. Unprocessed segmentation volume of dimensions *n*_*x*_,*n*_*y*_,*n*_*z*_. minv: *double*. Minimum allowed cell volume in 3D in *mm*^3^. maxv: *double*. Maximum allowed cell volume in 3D in *mm*^3^.

The allowable minimum and maximum cell volumes used in the running phase of the program are minvol and maxvol as seen in the struct variable displayed during runtime. These variables are derived from minv and maxv. In case of full 3D stacks containing the whole cell volume, the volume thresholds remain unchanged. However, for reduced 3D data sets, CELLSEGM computes modified values such that minvol <minv and maxvol <maxv. This ad-hoc system for modification of the cell volume applies to both segmsurf and segmct, and also the subroutines getminima and classifycells. The order of processing steps in segmsurf is described in the next sections.

#### Hessian ridge enhancement of segmentation image

A ridge enhancement increases the contrast of ridges compared to other structures. This process can be crucial for the success of a cell segmentation, as the plasma membrane for automated recognition becomes more strongly visible compared to other structures. In CELLSEGM a ridge enhancement is accomplished by ridgeenhhessian performing a Hessian ridge enhancement. Options are on (prm.filterridges = 1, *default*) or off (prm.filterridges = 0). The ridge enhancement is described in more detail in [23].

#### Detecting markers

**Figure 3:**
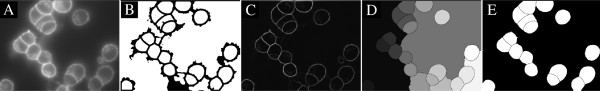
**Segmentation of cells in 2D using automatically detected markers in Example 5. A)** Raw surface stain, **B)** automatically detected markers, **C)** ridge enhanced surface stain, **D)** watershed image, **E)** detected cells.

For the analysis of high quality images with pronounced cell boundaries and limited endomembrane staining it is possible to recover the cells automatically from only the surface staining (cf. Figure 3). The markers are found automatically in getminima by adaptive thresholding, with the various steps explained in more detail in [23]. The available options are finding markers (i) automatically from the segmentation image (prm.getminima.method =’automated’, *default*), (ii) from the nucleus image using segmct (prm.getminima.method = ’nucleus’), or (iii) manually (prm.segmsurf.getminima.method = ’manual’ with the option minimacell and /or minima specified as argument to segmsurf, supplying the manually defined markers).

A nucleus channel is a powerful tool in order to generate markers inside cells (method (ii) above), in particular for datasets of medium or low quality. The signal from the nucleus stain outlines the nuclei of the cells, thus normally resulting in one distinct marker per cell. However, this can be violated when nuclei from two different cells are positioned in close proximity and therefore detected as one, or one cell can have several nuclei, as observed in cancer cells. Still, the nucleus method is powerful for huge datasets where the automated marker generation solely based on the surface stain is not successful, and where the manual definition of markers is too time consuming. CELLSEGM will find nucleus markers by setting prm.getminima.method = ’nucleus’, and by specifying the nuclues image imnucleus in the input. The nucleus markers are automatically detected using segmct, and therefore this function applies well to high-throughput data sets.

In given circumstances there is limited possibility to generate markers automatically, for instance due to poor data quality. Additionally, a nucleus staining may not be present due to previously acquired data lacking a nucleus channel, non-available equipment, or crosstalk between image channels. For these situations there exists an option in CELLSEGM to apply manually painted markers from a binary image where spatially connected components of "ones" define cell markers and "zeros" define background (method (iii)). This procedure is a substitute for the automated detection of markers (method (i)), or the nucleus based marker detection (method (ii)). The only restrictions for the manually assigned markers are that every marker entirely must be surrounded but not overlapped by the cell membrane, and there should be only one marker inside each cell. The positioning of the marker will normally not influence the segmentation performance. An exception occurs if the nucleus membrane is strongly stained. For these situations the markers should at least cover the area including the nuclear membrane signal in one image plane to enable a whole cell detection. The manual markers are applied by the minima and/or mimimacell option in segmsurf. At the same time, method must be set as prm.getminima.method = ’manual’. The minima option defines all markers, both for background and cells. In case minima is defined, getminima is not executed. The minimacell option defines the markers for cells only. In case only minimacell is defined, getminima is called to define the background markers. The cell markers, the minimacell image, is also used for classification of cells if prm.classifycells.method =’minimacell’. Preferably, both minima and minimacell are given, defining all markers, and exclusively cell markers, where minimacell must be a subset of minima.

Manual markers by prm.getminima.method = ’manual’ have priority over prm.getminima.method = ’nucleus’, if both are given.

#### Segmentation

A marker controlled watershed segmentation is applied to the previously smoothed and ridge enhanced segmentation image [24]. The segmentation will generate exactly one object covering and surrounding every given marker, where the boundaries of the objects are separating the markers from other markers. A watershed segmentation is preferably applied to an image where the boundaries of the target objects are ridge-like structures. The standard watershed algorithm has no inherent smoothing, but the demand for a regularization of the obtained surface is reduced by an initial smoothing of the segmentation image. The watershed segmentation generates a piecewise constant region for each given marker, representing the obtained segmentation, here referred to as the watershed image. An example is shown in Figure 3D.

#### Classification

In the segmentation process the watershed image is constructed where each integer value corresponds to one labeled region. It remains to distinguish between cell objects and non-cell objects (background) in the watershed image. A classification of the non-classified regions is carried out in classifycells. There are two methods available, defined by prm. ∗.method (prm. ∗. method = prm.segmsurf.classifycells.method here, for brevity). Classification thresholds can be assigned with respect to minimum cell volume min, maximum cell volume maxv, intensity inside cells prm. ∗.intincell, intensity on border prm. ∗.intborder, convexity of cell area prm. ∗.convexvarea, or convexity of border prm. ∗.convexborder. An object must fullfill all specified classification thresholds in order to be classified as a cell.

#### Classification based on thresholds

Predefined thresholds can be used for classification (prm. ∗.method = ’threshold’, *default*). By setting prm. ∗.propname = ’all’, the available features are minimum and maximum volume, normalized cell interior and boundary intensities, convex area and convex perimeter. Each of these thresholds can be specified in the parameter data struct. Fewer and selected classificators can be defined in the struct prm. ∗.propname as a cell array defining the property names as stated above.

#### Classification based on cell markers

If cell markers are available from manual markers and the minimacell option in segmsurf, or from nucleus markers, this information can assist in the cell classification, yielding a high degree of correct classifications (set prm. ∗.method = ’minimacell’). A region having a spatial overlap to a cell marker in the binary image minimacell is classified as a cell as long as the minimum and maximum volume is satisfied. These two additional classifiers are essential in case a cell region was merged to the background and became extraordinary large.

### Segmentation of stained nuclei and cytoplasmically stained cells - segmct

The level of complexity for segmentation of cytoplasmically stained cells depends on the density of cells and the signal homogeneity. A cytoplasmic staining is inappropriate if the aim is to distinguish between adjacent cells, since the boundaries between adjacent cells are not clearly visible by this type of staining. Instead, it is recommended to use a surface staining for this task, in combination with segmsurf. In CELLSEGM, segmct is essentially used for the segmentation of stained nuclei for marker generation in the watershed segmentation, where the name is derived from "CellTracker" probes.

#### Segmentation

The segmentation of stained nuclei or cytoplasmically stained cells is accomplished by segmct.

Syntax: segmct(im,minv,maxv)im: ${\text{double}}_{d_{x}\times d_{y}\times d_{z}}$. Unprocessed segmentation image where *d*_*x*_,*d*_*y*_,*d*_*z*_ is the image dimension. minv: *double*. Minimum cell volume in 3D in *mm*^3^. maxv: *double*. Maximum cell volume in 3D in *mm*^3^.

segmct has the option for several methods, as defined by the parameter struct prm as additional argument (type help cellsegm.segmct): *Adaptive thresholding* (prm.method = ’adth’) captures high intensity regions. It requires a large filter radius in order to capture whole cells, and is therefore slow for 3D data. The adaptive threshold is adjusted by prm.adth.adth. The filter radius is controlled by prm.adth.filtrad. *Iterative thresholding* (prm.method=’thrs’, *default*) applies a global thresholding until the lower cell volume limit is reached. The implicit thresholding value is specified by prm.thrs.th, and computed explicitly as a multiple of the threshold arising times graythresh with no arguments.

#### Splitting of cells

In the process for segmentation of stained nuclei or cytoplasmically stained cells, the detected objects are frequently incorrectly connected due to lack of strong edges between the objects. For improvement, splitcells can be run either separately after the segmentation or as a postprocessing step in segmct (prm.split = 1, *default*). splitcells applies the Euclidean distance transform of the binary segmentation image to find local maxima and thereby the cut around the maxima where the Euclidean distance is equal to the distance from another maximum [6]. The extent of splitting is adjusted by prm.splitth, becoming more pronounced for smaller values. The parameter prm.splitth is the second argument in imextendedmax. splitcells is implemented for 2D due to the functionality of the distance function, but it applies to 3D images section wise. The 2D plane for the splitting must be specified. As default setting, the plane for splitting is taken as one third height of the stack.

## High-throughput or batch segmentation

### Running a batch job - cellsegmentation

The CELLSEGM package is in particular designed for high-throughput experiments and is therefore the main processing feature. For this task, the algorithm cellsegmentation is used, processing all image stacks in a folder from given starting to ending indices. The input files must have the ordered names ‘stack1.mat’, ‘stack2.mat’, for all stacks, as prepared by readbioformat. For conversion of the data, see Section Image formats.

After conversion of the raw data, a batch job can be run using cellsegmentation. The segmentation job is executed through segmsurf or segmct, as described earlier. The tool cellsegmentation reads a parameter file for processing of the given data, as described in Section The parameter file of cellsegmentation. The use of a parameter file ensures reproducibility and documentation of the applied parameter settings. If a stack can not be loaded from disk, the program continues to the next stack, after printing an error message to the screen. cellsegmentation takes several arguments: Syntax: cellsegmentation(folder,sts,ste,pls,ple,minv,maxv,prmfile)

folder: *string* or cell array of *strings*_*n*×1_. The full path of the folders for processing either as a string or a cell array of strings, where *n* is the number of folders. sts: *double*. Numbering of starting stack. ste: *double*. Numbering of final stack. pls: *double* or *double*_*m*×*n*_. Starting plane of stack (row index) and folder (column index). ple: *double* or *double*_*m*×*n*_. Final plane for segmentation as for plsminv: *double*. Minimum cell volume in *mm*^3^. maxv: *double*. Maximum cell volume in *mm*^3^. prmfile: *string*. Full path to the parameter file. Default settings are used when the parameter file is empty (prmfile = []).

The variables pls and ple can be either scalars or matrices containing information about all stacks in all specified folders. Note that in case of pls being *double*_*m*×*n*_, missing values are filled out with NaN, resulting in no cell segmentation.

The processing results from cellsegmentation are stored in the same folder as where the data are located. If cellsegmentation is applied with manually given markers, there must exist Matlab .mat files with naming stack1-mask.mat, stack2-mask.mat, and so forth, each file containing two mandatory variables, minima and minimacell. These are binary images of the same dimension as the segmentation image, defining markers and cell markers, respectively.

#### The parameter file of cellsegmentation

The last input argument of cellsegmentation is the full path to a parameter file with user-defined settings for the segmentation. Undesignated parameters are assigned default values. Default values are specified in the help section of each routine by typing ‘help myfunc’. A parameter file may appear as in Example 2.

*Example 2.*prmfilenucleus

There is a distinct difference between the input variable pls and prm.segmplane as indicated in the parameter file. The input parameter pls indicates at which plane to cut the data for processing, where the excluded data is removed from any further analysis. The parameter specification prm.segmplane represents the starting plane, after cutting the data, from where to start the segmentation. The segmentation at this level is then copied down the array until the first plane. This option is useful when the signals in the lower planes are of reduced quality for segmentation.

**Figure 4:**
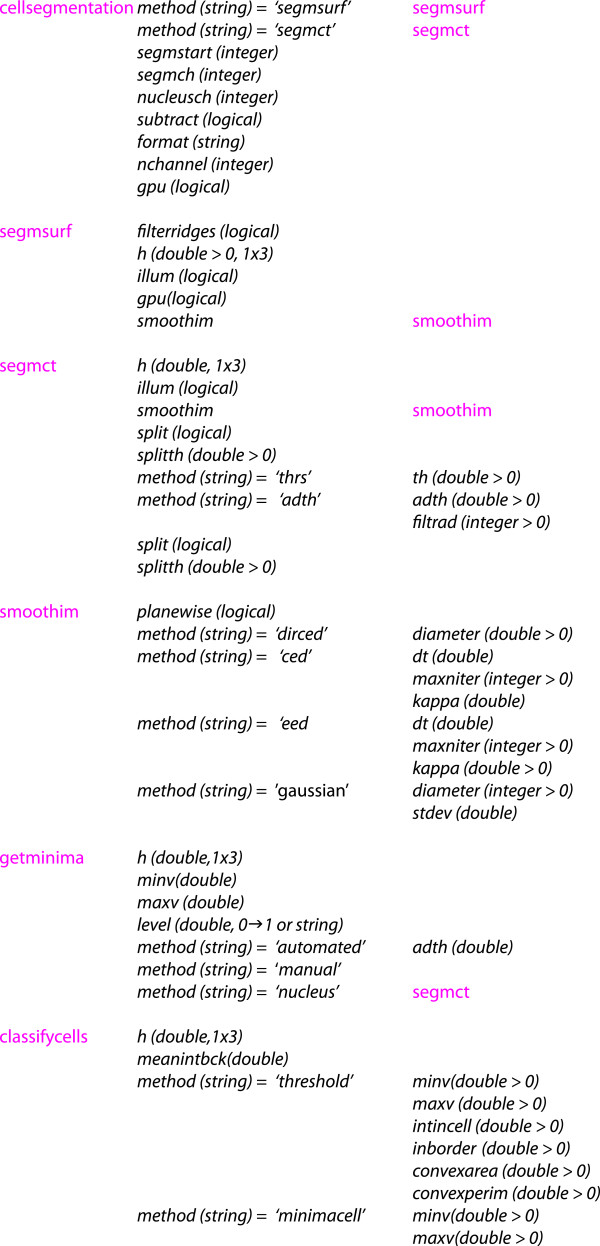
**A selection of available subroutines (magenta) and parameter settings (italic) in ****CELLSEGM ****related to the struct prm.** In brackets is the supported data type. For example, to set the threshold th in the nucleus method of segmsurf, with thresholding method thrs, follow the stream of methods segmsurf →getminima →nucleus →segmct →thrs, and assign it by prm.segmsurf.getminima.nucleus.thrs.th in the parameter file. On the other hand, if segmct is called upon from the command prompt, the same parameter setting is defined by prm.thrs.th.

The various levels of parameter settings are organized rigorously. For instance, consider the application of the functions segmsurf calling getminima, again calling segmct for constructing nucleus markers. Each of these functions has a legal set of parameter settings as specified in their individual help section. The segmentation method used in segmct is specified by, for instance, setting prm.method = ’thrs’, when executed from the command prompt. However, one can exploit the parameter settings in a hierarchical system from the top level in the parameter file. In the example above, the threshold for making nucleus markers in segmct is specified by setting prm.segmsurf.getminima.nucleus.thrs.th, composed of a set of keywords, related to prm.method in the various routines. The first part is the segmentation method, the second part getminima refers to the function, getminima. The third part, nucleus, refers to prm.method=’nucleus’ in getminima, the fourth part thrs refers to the segmentaton method prm.method=’thrs’ in segmct, and the last part refers to the threshold th in method = ’thrs’. By these means it is possible to specify a large number of parameters from a top level in the hierarchy, and also for each function individually. The overview of parameter settings is shown in Figure 4.

## Experimental results - a guided tour of CellSegm

In this section, the separate steps are explained in more detail and accompanied by comprehensive examples. The first two commands, clear all and close all are removed since they are repetitive. All given examples are included in the CELLSEGM package and can be executed from MATLAB by typing the name of the m-file in the command prompt (except from Example 1).

### Chemicals, procedures, and imaging protocols being used in the examples

For the examples presented in this work, Dulbecco’s modified eagle medium (DMEM), fetal calf serum (FCS), and wheat germ agglutinin Alexa Fluor 488 conjugate (WGA-AF-488) were purchased from Invitrogen Detection Technologies (Carlsbad, CA, USA), Hoechst staining (bisBenzimide H 33342 trihydrochloride) was purchased from Sigma-Aldrich (St. Louis, MO, USA). Microscopy-compatible 24-well plates were purchased from Greiner bio-one (Frickenhausen, Germany).

HeLa-Kyoto cells were cultured on microscopy-compatible 24-well plates in DMEM/10% fetal calf serum (FSC) with a final density of up to 35 000 cells/cm^2^, which corresponds to a confluent cell layer. Prior to cell segmentation, cells were fixed and stained by incubation in the following solutions for the indicated time-periods at room temperature: phosphate buffered saline (PBS), 1 min; paraformaldehyde (4%)/ sucrose (4%)/PBS, 35 min; NH_4_Cl (50 mM)/PBS, 2 min; PBS, 1 min; wheat-germ-agglutinin-Alexa-Fluor 488 (500 ng/ml)/ Hoechst-staining 33342 (4 *μ*g/ml)/PBS, 10 min; PBS, 1 min; paraformaldehyde (4%)/ sucrose (4%)/PBS, 10 min; NH4Cl (50 mM)/PBS, 2 min; PBS, 1 min; PBS, 1 min. The resulting fixed and stained cultured cells proved to be suitable for microscopical image acquisition of segmentation quality for at least one week. PC12 (pheochromocytoma 12) cells were cultured in 10% horse serum, 5% fetal calf serum.

Immunohistochemical staining for CD44 and p53 was done on formalin-fixed paraffine embedded human skin biopsy tissue, showing epidermis and dermis, including part of a hair follicle and sebaceous gland. Tissue slides were dried 30 min at 70°C. Deparaffinised 3 *μ*m sections were double-stained for CD44 and p53 sequentially, in two steps. Antigen retrieval was performed by incubation in a pressurized heating chamber (Pascal; Dako, Glostrup, Denmark) at 121°C for 30 sec in Tris-EDTA buffer (pH 9). P53 was detected by the monoclonal antibody clone DO-; DAKO (M7001) diluted 1:1000 in TBST (pH 7.4), incubated for 60 min at room temperature (RT). Detection system MACH-3 HRP (Biocare Medical (M3M530L)). MACH-3 mouse probe - incubation 20 min/RT. MACH-3 mouse HRP polymer - incubation 20 min/RT. Blocking: 3% hydrogen peroxide for 5 min. After colour development in DAB (DAKO (K3468) incubation 10 min/RT) the slides were rinsed in running tap water and then placed in preheated (100°C) Tris EDTA buffer (pH 9) for 2 min (modified antigen retrieval). CD44 was detected by the monoclonal antibody G44-26 (BD Biosciences, San Jose, CA). The antibody was diluted 1:100 in TBS antibody diluent (pH 7.4), incubated for 60 min at RT. Detection system MACH-2 AP (Biocare Medical (MALP521G)), MACH-2 AP polymer - incubated 30 min/RT. Colour development in Vulcan fast red (Biocare medical (FR 805H)) incubation 10–15 min/RT. The sections were counterstained with Harris’s hematoxylin (Histolab Products, Gothenburg, Sweden) for 30 sec and then dehydrated in alcohol, cleared in xylene, and coverslipped using a Mountex permanent mounting medium (Histolab Products).

Cells were imaged with a Leica confocal SP5 microscope in the resonant scanner mode; excitation 430 and 488 nm; zoom 1.7; pinhole airy 1; 40x 1.25NA oil immersion objective; 512x512 pixel; z-distance 1.01 *μ*m; line-average 16; offset -1; gain 1000 V. Examples ??, ?? and ?? show PC12 cells and HeLa Kyoto cells are displayed in the remaining examples. Tissue was imaged with a 63x 1.4NA oil immersion objective; 1024 × 1024 pixel; z-distance 0.29 *μ*m, line-average 64; offset -1; gain 900 V.

### Segmentation of surface stained cells and segmsurf

#### Smoothing of the segmentation image

**Figure 5:**
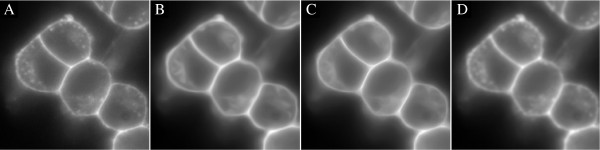
**Smoothing of PC12 cells in 2D by Example 3. A)** Raw surface stain, **B)** smoothing by coherence enhancing diffusion (method = ’ced’), **C)** directional coherence enhancing diffusion (method = ’dirced’), and **D)** Gaussian smoothing (method = ’gaussian’). The sharpness is better preserved by the anisotropic filters **(B and C)**, which makes them more suitable for the enhancement of surface stained cells.

Example 3 demonstrates a smoothing of surface stained cells in 2D using smoothim. The output from the code is seen in Figure 5. The anisotropic filters better preserve the high-signal characteristics on the cell boundaries than the Gaussian smoothing, and are therefore better suited for smoothing as a preprocessing step to segmentation. The routine show is a visualization tool, where the first argument is the 2D or 3D image to visualize, and the second argument specifies the figure number.

*Example 3.*surfstain_smoothing_2D

**Figure 6:**
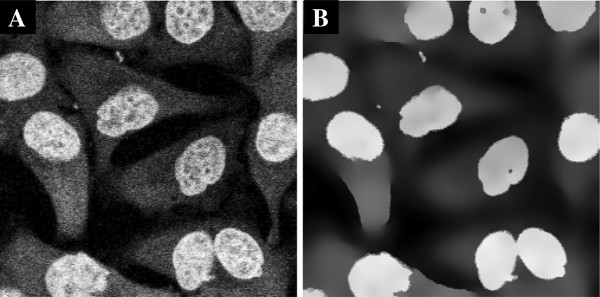
**Smoothing of stained nuclei of Hela-Kyoto cells in 2D by Example 4. A)** Raw nuclei stain, **B)** smoothing of A by edge enhancing diffusion (method = ’eed’). After edge enhancing diffusion the image becomes more piecewise constant and better suited for segmentation.

Example 4 shows smoothing of stained nuclei by edge enhancing diffusion (method = ’eed’). This option is most useful for objects that are not characterized by ridges (i.e. surface stained cells) but rather by high-intensity regions like stained nuclei or cytoplasmically stained cells. The output from the code is seen in Figure 6. The resulting edge enhanced image is better suited for segmentation as similar structures are similar in intensities and surrounded by sharp gradients, and therefore more manageable in the further processing.

*Example 4.*nucleistain_smoothing_2D 

#### Automatically defined markers

**Figure 7:**
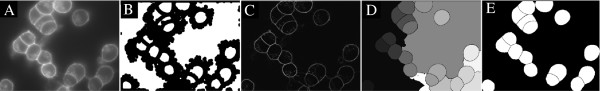
**Segmentation of cells in 3D using automatically detected markers in Example 6. A)** Raw surface stain, **B)** automatically detected markers, **C)** smoothed and ridge enhanced surface stain, **D)** watershed image, **E)** detected cells.

**Figure 8:**
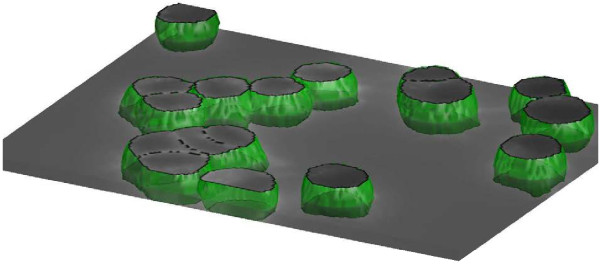
**3D view of the segmentation in Example 6.** The obtained segmentation is truly a 3D segmentation field. For visualization purposes the cells have been cut at plane 20.

Example 5 shows a 2D cell segmentation of WGA-AF-488 stained PC12 cells imaged with a Zeiss wide field microscope, where the markers are automatically generated (Section Detecting markers, prm.getminima. method = ’automated’). The output of Example 5 is seen in Figure 3, where all cells have been well outlined. Generally speaking, a 2D segmentation can be more challenging than 3D due to the lack of 3D spatial connectivity information, in particular for the background. The shortcoming of information can heavily influence the automatic creation of markers. Still, a 2D segmentation can be highly useful for fast parameter tuning of the algorithm, and to get an impression of the efficiency on a particular type of data. The segmentation of the same data set in 3D is demonstrated in Example 6 and Figures 7 and 8.

*Example 5.*surfstain_2D

*Example 6.*surfstain_3D

#### Markers from nucleus channel

**Figure 9:**
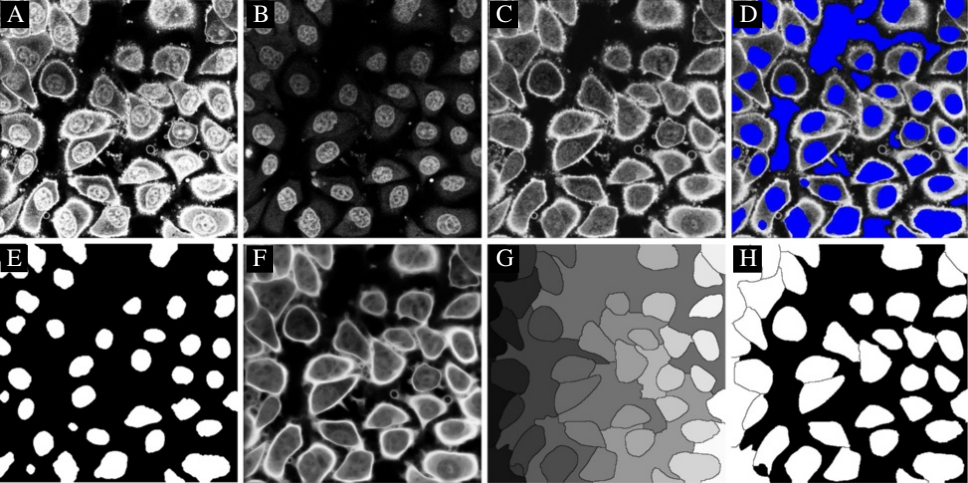
**Segmentation of cells using nucleus markers in 2D from Example 7, executed for plane five in the image stack. A)** Raw surface stain, **B)** raw nucleus stain, **C)** surface stain minus nucleus stain, **D)** markers (blue) derived from the nucleus stain superimposed onto the surface stain, **E)** cell markers, **F)** smoothed segmentation image, from **C**, **G)** watershed image, **H)** detected cell areas.

The examples for finding markers in the nucleus channel are taken from the images of Kyoto HeLa cancer cells, acquired on a confocal Leica SP5 microscope. There are two image channels, the WGA-AF-488 (variable imsegm) and Hoechst (variable imnucl). We subtract the nucleus channel from the WGA-AF-488 channel to reduce the influence of cross talk from the nucleus channel into the WGA-AF-488 channel, occurring from simultaneous imaging. Without this subtraction the nucleus may be classified as the whole cell. For the subtraction we first convolve both images with a Gaussian, otherwise, the impact of noise is substantial. Example 7 demonstrates segmentation of 2D surface stained cells with nucleus markers. These images contain a substantial amount of unidentifiable structures in the cells resembling ridges, and a ridge filtering is therefore not feasible since it will generate artificial structures. We use splitting of nucleus markers since this can split incorrectly fused cell nuclei into their separate parts. The output from the code is seen in Figure 9, where all cells have been found.

*Example 7.*surfstain_and_nucleus_2D

**Figure 10:**
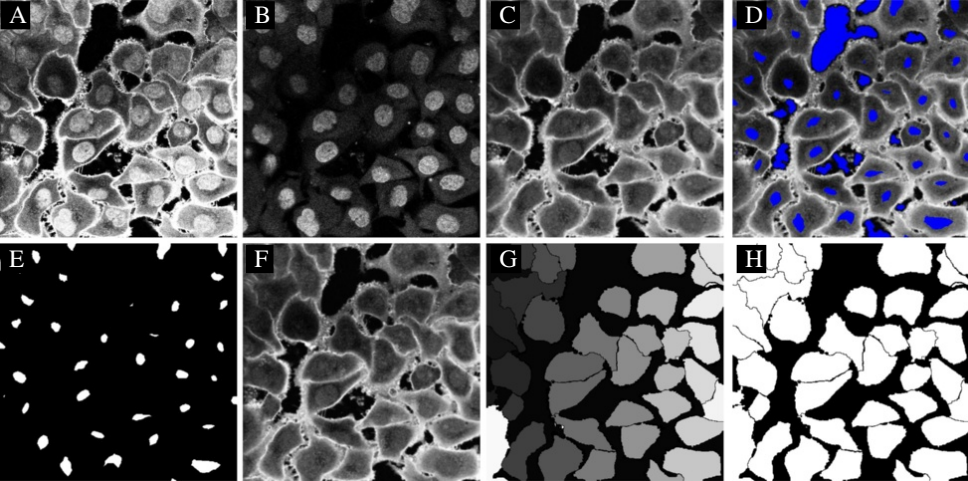
**Segmentation of cells using nucleus markers in 3D from Example 8, visualized for plane two. A)** Raw surface stain, **B)** raw nucleus stain, **C)** surface stain minus nucleus stain, **D)** markers (blue) from nucleus stain superimposed on the surface stain, **E)** cell markers, **F)** smoothed input image, from **C**, **G)** watershed image, **H)** detected cells. All cells have been detected.

Example 8 is a full 3D segmentation of the same data as for Example 8. The output from the code is seen in Figure 10. Here, no smoothing of the input image was applied, to demonstrate that the boundaries become slightly oscillatory. Without the availability of the nucleus markers, the blind segmentation task is considerable.

*Example 8.*surfstain_and_nucleus_3D

#### Manually defined markers

**Figure 11:**
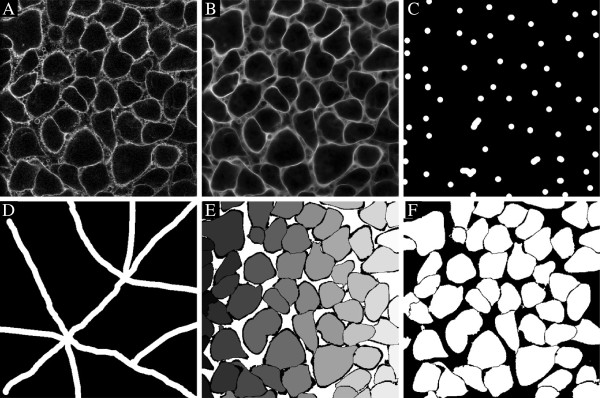
**Segmentation of cells using manually drawn markers in 3D from example 9, visualized for plane five. A)** Raw surface stain, **B)** smoothed surface stain used for segmentation, **C)** cell markers drawn manually, **D)** background markers (in an imaging plane other than the cell markers), **E)** watershed image, **F)** detected cells.

Example 9 is a segmentation of surface stained cells with manually "painted" markers. IMAGEJ (http://rsb.info.nih.gov/ij) was used to define the markers, but any drawing tool can be applied where the markers can be exported to a multiple .tif file. Manually defined markers can for instance be useful when the segmentation is applied to old data files where the nucleus channel was not acquired, or for new data where all available imaging channels are needed for biological quantification. The manual painting requires one seed within each cell and is therefore significantly less labor intensive than manual segmentation. Still, the application of manual markers for high-throughput data sets is costly with respect to time consumption. The output from Example 9 is seen in Figure 11, and the segmentation was successfull. This example was only executed in 3D since the manual markers were assigned at different levels in 3D.

*Example 9.*surfstain_and_manual_3D

### Segmentation of cytoplasmically stained cells or stained nuclei using segmct

**Figure 12:**
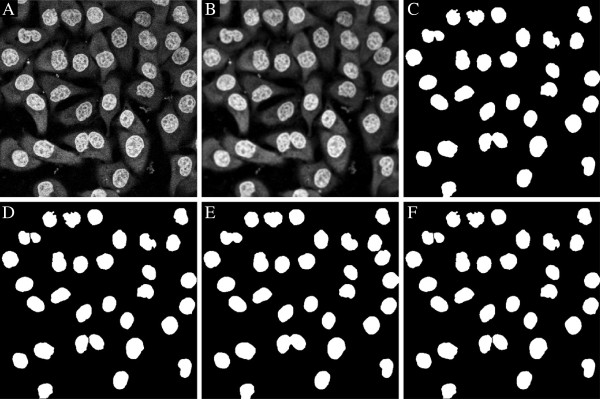
**Segmentation of Hoechst stained Hela-Kyoto nuclei in 2D using **segmct **from Example 10. A)** Input image showing stained nuclei, **B)** after edge enhancing diffusion, **C)** segmentation by adaptive thresholding (prm.method = ’adth’) without splitting of cells, and **D)** with splitting of cells. Note that the connected nuclei are now disconnected. **E)** Segmentation by iterative thresholding (prm.method = ’thrs’) without splitting, and **F)** after splitting. Both methods are successfull.

A segmentation of cytoplasmically stained cells or stained nuclei can be biologically useful. Example 10 is a segmentation of Hoechst stained nuclei, by all three available methods in segmct. The results are shown in Figure 12, and all three methods are successfull. They all apply a splitting algorithm to split objects that are wrongly connected. This splitting algorithm is described in splitcells and relies on the Euclidean distance function to separate the objects. The splitting parameter prm.splitth in splitcells controls the amount of splitting.

*Example 10.*nucleistain_2D

**Figure 13:**
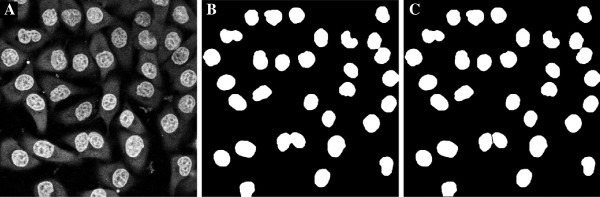
**Segmentation of Hoechst stained nuclei in 3D using **segmct**, from Example 11. A)** Raw nucleus stain, **B)** segmentation with iterative thresholding (prm.method = ’itth’) without splitting of cells, **C)** after splitting of cells. Note that after splitting several connected nuclei are disconnected into their separate compartments.

The next example demonstrates a 3D segmentation of stained nuclei from the previous example, only including the option prm.method = ’thrs’ since the adaptive thresholding with prm.method = ’adth’ has substantial CPU times in 3D. The output from the code is seen in Figure 13, where the nuclei are successfully outlined. The results are computed in 3D but only visualized in 2D.

*Example 11.*nucleistain_3D

### Batch processing in CELLSEGM

A batch processing job is the major feature in CELLSEGM and can be conducted by cellsegmentation as described in Section Running a batch job - cellsegmentation. The parameters in use are defined in a parameter file given as an argument to cellsegmentation, or as an input struct. The input struct has the highest priority. In Example 12, two experimental conditions are processed for a demonstration of a larger job. Each condition contains two 3D stacks. The data are Hoechst stained nuclei and WGA-AF-488 stained cells. The preprocessed data are also available in ‘data/condition1-preprocessed’ and ‘data/condition2-preprocessed’, included in the CELLSEGM package. The parameter file in use is printed in Example The parameter file of cellsegmentation.

*Example 12.*surfstain_and_nucleus_cellsegmentation_3D

**Figure 14:**
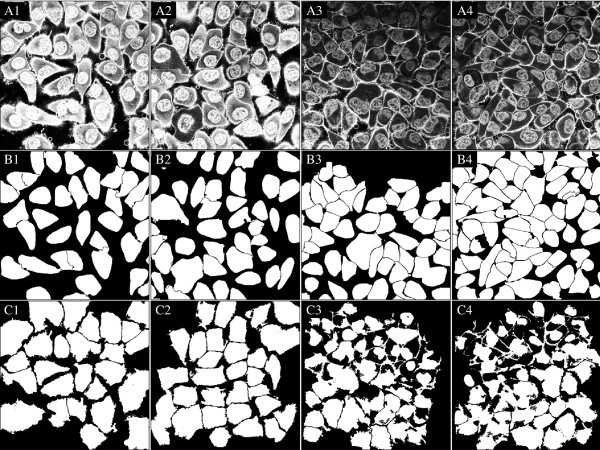
**A batch processing of two data sets from two experimental conditions, as described in Example 12.** The data is visualized for plane seven. **A1-A4)** Data set one and two in the two conditions. **B1-B4)** Segmentation using CELLSEGM. **C1-C4)** Segmentation using CELLPROFILER. For the strongly stained cells, CELLPROFILER provides a larger segmentation than CELLSEGM. For the weakly stained cells, CELLPROFILER is missing large cell fractions compared to CELLSEGM, probably due to uneven illumination. However, a correction of the uneven illumination pattern uneven did not improve the results (data not shown). For visualization, the objects segmented in CELLPROFILER where eroded by one voxel to highlight the contours.

The segmentation is shown in Figure 14. A segmentation of the same data sets was also performed in CELLPROFILER for quantitative comparison (see Section Quantitative analysis of segmentation performance).

An automated analysis is normally not fully automatic with respect to user-intervention. The results of a segmentation, or at least major parts of them, must be quality-checked by the end-user. This makes it possible to judge and decide whether a satisfactory segmentation has been obtained, or whether a rerun with new parameter settings must be conducted. The results from cellsegmentation can be visualized by viewsegm. It takes four arguments:

start: *integer*. Numbering of first stack. stop: *integer*. Numbering of last stack ch1: *integer*. The order of the first visualization channel (1,2,...) ch2: *integer*. The order of the second visualization channel (1,2,...)

Two windows will appear; the control panel and the image panel. The control panel allows the user to move up (‘Up’) and down (‘Down’) in the stack, to proceed to next (‘Next’) or previous (‘Previous’) stack, to manually enter the frame number (‘Frame’), or to print the classification data (‘Classification’). The latter is useful for parameter tuning. It reveals, by clicking on the image, why an object was accepted or rejected as a cell.

### Quantitative analysis of segmentation performance

In order to quantitatively evaluate the performance of CELLSEGM, the four data sets in Section Batch processing in CELLSEGM were independently manually segmented in IMAGEJ by two experts in cell biology (T.K. and D.M.F.). All manual delineations were performed planewise, summing up to a 3D volume, and then compared to the automated segmentations, as well as compared to each other. The two manual observers independently found 237 (T.K.) and 240 (D.M.F.) cells in the four datasets. We have adopted the approach in [21] and [23] where the coefficient for success is expressed as the fraction of intersection and union

(1) $$C_{1}=\frac{A\cap B}{A\cup B}$$

for two given segmentations *A* and *B*. This coefficient is more conservative than the Dice coefficient. *C*_1_ contains no information with respect to over- or under-segmentation, and we have added two expanded coefficients *C*_2_,*C*_3_ as described in [23],

(2) $$C_{2}=\frac{A\cap B}{A\setminus B+A\cup B},C_{3}=\frac{A\cap B}{B\setminus A+A\cup B}$$

where *A*∖*B* means the elements in *A* not contained in *B*, and vice versa. A high value of *C*_2_ and a low value of *C*_3_ corresponds to a an under-segmentation of *A* compared to *B*, and a high value of *C*_3_ and a low value of *C*_2_ corresponds to a an under-segmentation of *B* compared to *A*. Further, to ensure a one-to-one correspondance of segmented regions, a region in one segmentation can map to at most one region in the other segmentation. Using the framework in Hodneland et al. [23] we ensure the optimal one-to-one correspondance of various regions. Additionally, based on the number of cells present in a specific image, each evaluation coefficient was normalized to the total number of cells available for the evaluation. This normalization ensures an unbiased coefficient, independent of the number of cells in each image. The manually segmented data sets and those segmented by CELLSEGM were voxelwisely compared according to the evaluation scheme using binary and not probabilistic segmentations. A coefficient *C*_*i*_ is always between zero and one, *C*_*i*_→0 is a poor segmentation and *C*_*i*_→1 is associated with better segmentation for all *i*={1,2,3}.

The results from the comparison are presented in Table 2, where the two independent observers had an agreement of *C*_1_=0.8238. Observer 2 was more conservative than observer 1 as indicated by *C*_2_<*C*_3_. The best value of *C*_1_ for CELLSEGM was *C*_1_=0.7080. The inter-observer disagreement 1 - C _*i*_(O_1_-O_2_) was subtracted from the automated segmentations to obtain a normalized evaluation coefficient *C*_*i*,*n*_ reflecting the disagreement exceeding the disagreement between the expert observers. By this subtraction, CELLSEGM had an agreement level with the manual observers of *C*_1,*n*_= {0.8534,0.8842}.

**Table 2:** **Quantitative comparison of volumetric segmentation accuracy between two manual observers (O**_**1**_**,O**_**2**_**), ****CELLPROFILER ****and ****CELLSEGM**

**Comparison/Coefficient**	***C***_***1***_	***C***_***2***_	***C***_***3***_	***C***_** 1*,n***_	***C***_** 2*,n***_	***C***_** 3*,n***_
O_1_-CellSegm	0.6772	0.7701	0.7302	0.8534	0.9187	0.7685
O_2_-CellSegm	0.7080	0.7690	0.7992	0.8842	0.9176	0.8375
O_1_-CellProfiler	0.1161	0.2961	0.1439	0.2923	0.4447	0.1822
O_2_-CellProfiler	0.1238	0.3060	0.1608	0.3000	0.4546	0.1991
O_1_-O_2_	0.8238	0.8514	0.9617	1.0000	1.0000	1.0000

The columns are the evaluation coefficients **C**_**i**_**, i = 1, 2, 3**, as described in (1) and (2). The normalized evaluation coefficients **C**_**i**,**n**_ are also presented, arising after subtracting the inter-observer variability from **C**_**i**_. CELLSEGM has considerably higher success rates than CELLPROFILER.

A segmentation of the same data sets was also performed in CELLPROFILER. The workflow was detection of primary objects (nuclei), followed by detetion of secondary objects (whole cells). An illumination correction was tried but abandoned due to lower success rates. We explored all available segmentation methods for secondary objects in CELLPROFILER (Propagation, Watershed-Gradient, Watershed-Image, Distance-N, Distance-B) and we here report the best results, which were obtained by "Propagation". The results from the segmentation evaluation are reported in Figure 14. CELLPROFILER had much lower performance of *C*_1,*n*_={0.2923−0.3000} for the normalized evaluation coefficient. The observation of *C*_2,*n*_>*C*_3,*n*_ for both observers reveals that both manual observers were more conservative than CELLPROFILER. We also did a volumetric independent quantitative analysis of the over- and under-segmentation of CELLPROFILER and CELLSEGM, by counting the falsely fused, splitted, positive and negative cells, and reporting the counted events (Table 3). CELLSEGM had lower values of falsely fused, false negative, and false positive cells, and CELLPROFILER had lower values of falsely splitted cells. The cells that were falsely splitted by CELLSEGM were binuclear cells. On overall, CELLSEGM has an improved segmentation accuracy compared to CELLPROFILER.

**Table 3:** **Quantitative comparison of segmentation accuracy between ****CELLPROFILER ****and ****CELLSEGM ****as measured by falsely fused, splitted, positive and negative cells for all four data sets**

**Comparison/**	**Falsely fused**	**Falsely splitted**	**False positive**	**False negative**
O_1_-CellSegm	15	13	1	1
O_2_-CellSegm	6	11	2	8
O_1_-CellProfiler	72	4	0	49
O_2_-CellProfiler	62	0	0	56

CELLPROFILER has much more falsely fused cells and false negative cells than CELLSEGM, although CELLSEGM has more falsely splitted cells. The latter is mostly due to cells having two nuclei, which is not uncommon for cancer cell lines. The false positive and false negative rates are very low for CELLSEGM. The robustness of CELLSEGM is greatly improved when the nucleus approach is applied, where a cell is initiated only if a valid nucleus marker exists.

### Automated segmentation of a tissue sample

**Figure 15:**
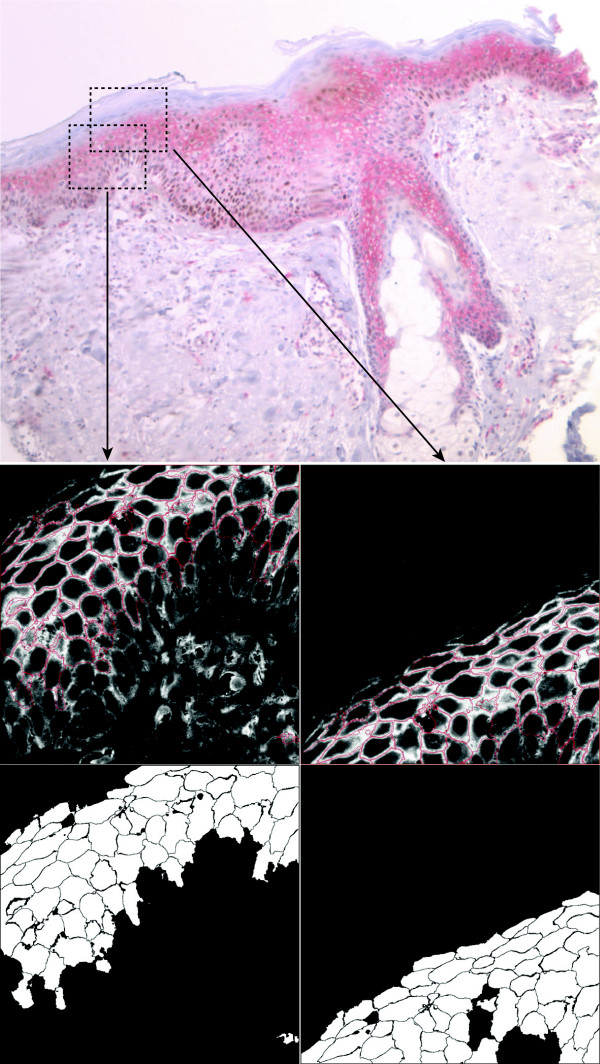
**Segmentation of a paraffine embedded human skin biopsy.** Upper row: Light microscopical image of the whole sample, visible are the layers of the epidermis and dermis, including a part of a hair follicle. Stained are CD44 (red) and p53 (brown). Middle row: One plane of a 3D confocal fluorescence image stack of CD44 (VulcanRed; white), overlaid with the segmentation from CELLSEGM (red). For visualization purposes, the contours were dilated with a structural element of one pixel radius, and then closed with a structural element of four pixel radius. Lower row: Segmentation results using CELLSEGM (no dilation and no closing here). The segmentation is essentially confined to the cells expressing the marker at the plasma membrane to a sufficient amount.

To demonstrate the versatility of CELLSEGM we also accomplished an automated, 3D segmentation of cells of a human skin biopsy. This application demonstrates the potential of CELLSEGM in clinical biomarker analysis. In this case CD44 has been stained by immunocytochemistry in a human skin slice and this has been used to automatically segment the cells of the epidermis. This sample could be segmented successfully even without nuclear markers. The procedure for segmentation is given in Example 13. For these data the segmentation was executed by automatically finding markers for the watershed segmentation. This option is available by setting prm.segmsurf.getminima.method = ’automated’. For tissue slices the optimal section for finding markers is often the plane of highest intensities because this often corresponds to the level where the tissue is most complete. This option is enabled by prm.segmsurf.getminima.level = ’strong’. A manual setting of the background used for cell classification was also chosen here, by specifying prm.segmsurf.classifycells.meanintbck = 1. This option is particularly useful for situations where the image contains hardly any background (non-cell) regions. A satisfying segmentation result was obtained, as shown in Figure 15.

*Example 13.*tissue_3D

## Discussion and conclusions

The automated analysis of single cells in huge datasets has a large potential in the screening of high-throughput microscopy-generated data. In particular, our segmentation of surface stained cells enables a whole cell segmentation of both single and clustered cells. We have demonstrated the performance and versatility of CELLSEGM by application to a wide range of imaging examples related to cell lines. It has proven to be a powerful tool for whole cell segmentation, also for clustered and confluent cell cultures. It can handle 2D as well as 3D datasets, and it has integrated an option for nucleus markers in the analysis. This option represents a substantial advantage compared to a ‘nucleus-blind’ segmentation, where the datasets must be of high quality in order to have successful results. Additionally, we have shown that it can be used for the segmentation of human biopsy tissue samples. However, one must consider that biopsy samples have highly varying characteristics depending on the cell type, the tissue cell organization, the staining, and the method of preparation. Thus, many samples are challenging to segment, and one can hardly make general statements with regard to the overall segmentation capacity of CELLSEGM applied to such samples. However, if the sample has homogeneously distributed and high-intensity signals on the cell boundaries, we have shown that CELLSEGM is likely to perform well. Therefore, if CELLSEGM cannot be used to segment every tissue or sample, but for the ones it can be used, it can have substantial advantages in terms of less biased, high-troughput and reproducible data-analysis.

In this respect, we see CELLSEGM as a considerable accretion to existing software, as it offers a collection of programs (Matlab functions) that can be executed from the command-line to perform cell segmentation of surface stained cells. The scripting modus in CELLSEGM provides large flexibility for the user, where the batch processing tool cellsegmentation as well as the separate programs can be used independently. These properties of CELLSEGM turn it into a practical solution for biologists who wish to program their own post-processing modules. Additionally, the MATLAB environment is a flexible programming interface with much functionality for interfering with the existing routines in CELLSEGM. As a pivotal advantage to flow-cytometry, with CELLSEGM, tissues or cell cultures cannot only be investigated with regard to the distribution of markers in a cell population, but it preserves the cells constellations in space. Therefore, we can imagine putative applications not only in cell cultures, but also in, for example, developmental research on small organisms as drosophila or zebrafish, or standardized pathological diagnosis routines.

Post-processing tools are presently not included in the CELLSEGM suite, and such toolbox would represent a useful extension for the future. At the present stage of development, users must employ external tools like Fiji / ImageJ or make further use of the MATLAB technical computing language and interactive environment for algorithm development of tailored post-processing and data analysis. In order to facilitate such developments, the segmentation in CELLSEGM can be exported as multiple.tif files and can therefore easily be imported into other software for further analysis. In this context, we encourage CELLSEGM users to share their post-processing code or plug-ins at our website for common use. Also, user experiences and suggestions for new functionality are most welcome. This will guide further developments of CELLSEGM.

In order to evaluate the performance of CELLSEGM we chose CELLPROFILER as a comparative open-source software. CELLPROFILER is a well established tool for segmentation of cells within the cell biology community. The results of the thorough comparison are presented in Table 2, showing a superior performance of CELLSEGM. The discrepancy of segmentation success reflects the different applicability of CELLPROFILER and CELLSEGM. CELLSEGM aims at segmenting the cells at the crest of the plasma membrane signal, while CELLPROFILER attempts to segment the cells at the outer boundaries, resulting in highly different volume measurements. Therefore, we also did an evaluation based on falsely fused, falsely splitted, false positive and negative cells, which is a volumetric independent analysis. Also here CELLSEGM had an overall better performance than CELLPROFILER, except from the falsely splitted cells where CELLSEGM had a tendency to split cells with a double nucleus into two fractions, since both nuclei were used as markers for the watershed segmentation.

In this respect it should be noted that CELLPROFILER produced a high-performing segmentation of our stained nuclei (data not shown).

Thus, CELLSEGM represents an independent tool for a type of cell segmentation which is currently not well supported in CELLPROFILER, and CELLSEGM could therefore be promoted for the segmentation of surface stained cells and other samples with similar characteristics.

The motivation and development of CELLSEGM is much in line with current trends in biology regarding acquisitions of high-content, high-throughput imaging data and the increasing demand for quantitative analysis. Only by integrating flexible and targeted software tools with efficient computing, such datasets can be turned into valuable biological information and generation of new hypotheses and experiment designs with optimal use of human resources and expertise.

## Competing interests

The authors declare that they have no competing interests.

## Authors’ contributions

EH did the implementation and development of CellSegm and writing of the paper. TK planned and developed the underlying experiments. She translated the life-sciences requirements to the programmer during the whole process of program development and wrote the biological parts of the article. TK and DF performed the image acquisition referred to in the examples and the underlying development of the nucleus approach for markers. They also created the manual segmentations for quantitative evaluation. H-HG planned the frame of the project and provided input to the software functionality in the early phase of development. AL took part in the development of the algorithms and in the writing of the manuscript. All authors read and approved the final manuscript.
